# Preclinical evaluation of the Hsp90 inhibitor SNX-5422 in ibrutinib resistant CLL

**DOI:** 10.1186/s13045-021-01039-9

**Published:** 2021-02-24

**Authors:** Timothy L. Chen, Bonnie Harrington, Jean Truxall, Ronni Wasmuth, Alexander Prouty, Shelby Sloan, Amy M. Lehman, Deepa Sampath, Eric Orlemans, Robert A. Baiocchi, Lapo Alinari, John C. Byrd, Jennifer A. Woyach, Erin Hertlein

**Affiliations:** 1 Division of Hematology, Department of Internal Medicine, The Ohio State University, 462 OSUCCC, 410 W 12th Avenue, Columbus, OH 43210 USA; 2 Department of Pathobiology and Diagnostic Investigation, College of Veterinary Medicine, Michigan State University, East Lansing, MI USA; 3 Center for Biostatistics, Department of Biomedical Informatics, The Ohio State University, Columbus, OH USA; 4 Esanex Inc., Indianapolis, IN USA; 5 Division of Pharmaceutics, College of Pharmacy, The Ohio State University, Columbus, OH USA

**Keywords:** Chronic lymphocytic leukemia, Hsp90, BTK

## Abstract

B-cell receptor (BCR) antagonists such as the BTK inhibitor ibrutinib have proven to effectively target chronic lymphocytic leukemia (CLL) tumor cells, leading to impressive response rates in these patients. However patients do still relapse on ibrutinib, and the progressive disease is often quite aggressive requiring immediate treatment. Several strategies are being pursued to treat patients who relapse on ibrutinib therapy. As the most common form of relapse is the development of a mutant form of BTK which limits ibrutinib binding, agents which lead to degradation of the BTK protein are a promising strategy. Our study explores the efficacy of the Hsp90 inhibitor, SNX-5422, in CLL. The SNX Hsp90 inhibitor was effective in primary CLL cells, as well as B-cell lines expressing either BTK wild type or C481 mutant BTK, which has been identified as the primary resistance mechanism to ibrutinib in CLL patients. Furthermore the combination of SNX-5422 and ibrutinib provided a remarkable in vivo survival benefit in the Eμ-TCL1 mouse model of CLL compared to the vehicle or single agent groups (51 day median survival in the vehicle and ibrutinib groups versus 100 day median survival in the combination). We report here preclinical data suggesting that the Hsp90 inhibitor SNX-5422, which has been pursued in clinical trials in both solid tumor and hematological malignancies, is a potential therapy for ibrutinib resistant CLL.

**Supplementary Information:**

The online version contains supplementary material available at 10.1186/s13045-021-01039-9.

To the editor,

In the front-line setting, agents targeting B-cell receptor (BCR) signaling have shown extremely promising results, and are an option for initial therapy in patients with Chronic Lymphocytic Leukemia (CLL) [1]. For patients with relapsed CLL the median progression free survival in CLL patients treated with ibrutinib was 44.1 months, with a cumulative overall response rate of 91%. Nevertheless, ibrutinib was discontinued in 37% of CLL patients due to progressive disease [2], and patients who relapse on ibrutinib progress quickly and have poor overall survival [3]. Patients who progress on BTK inhibitors which rely on the C481 binding site develop frequent mutations in *BTK* (C481S) which circumvents BTK inhibition in ~ 85% of cases [4]. Therefore combinatorial approaches to target mutant BTK could eliminate the mutant clone allowing the patient to continue on ibrutinib.

One promising clinical strategy in patients with resistant CLL is Hsp90 inhibition to target the BTK protein. Esanex Pharmaceuticals developed a novel Hsp90 inhibitor, SNX-5422 (the prodrug of SNX-2112) which has been safely tested in multiple phase I studies in solid tumors and hematological malignancies [5–7]. In treatment-naïve primary CLL cells we see reduced proliferation with as low as 0.1uM SNX-2112 (Fig. 1a) including CpG stimulated primary CLL cells which mimics the natural stimulation of the tumor microenvironment (Fig. 1b). Furthermore we found that downstream mediators of BCR signaling, BTK and AKT, are consistently down-regulated in all patient samples examined (Fig. 1c). Furthermore while ibrutinib is able to reduce BTK autophosphorylation at Y223 in cells expressing wild type BTK protein, cells expressing C481S mutated BTK [8, 9] are resistant (Fig. 1d). However we see a reduction in both phospho and total BTK with 0.1uM SNX-2112 in both WT and C481S BTK cell lines.

**Fig. 1:**
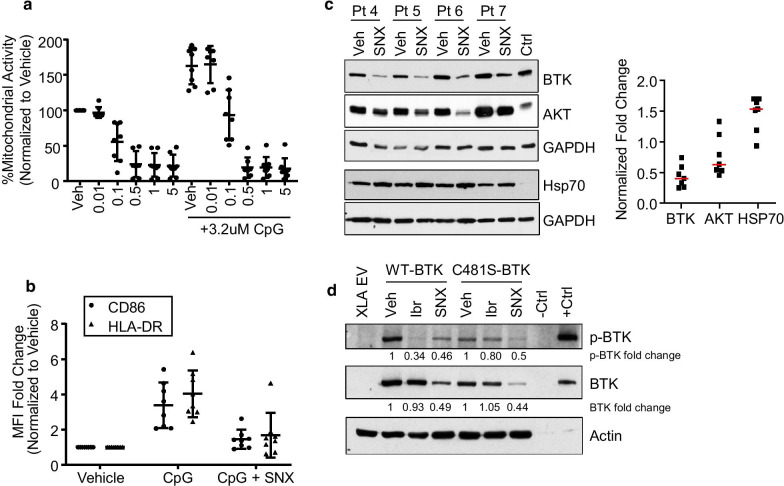
**a** CLL B-cells (N = 8) were plated in 96-well plates at 400,000 cells per well. Cells were treated with either vehicle, 0.5uM SNX-2112, or 3.2uM CpG + 0.5uM SNX-2112 for 48 h followed by addition of MTS reagents and samples were read at 490nm. **b** CLL B-cells (N = 8) were treated with either vehicle, 3.2uM CpG, or 3.2uM CpG + 0.5uM SNX-2112. CD19+ and live cells were stained and analyzed by flow cytometry for HLA-DR and CD86 surface expression. **c** CLL B-cells isolated from the peripheral blood of patient samples (N = 7) were treated with vehicle or 0.5uM SNX-2112 for 16 h. Whole cell lysates were isolated and immunoblots performed to determine total levels of BTK, AKT and Hsp70 protein, as well as the loading control GAPDH (left). Immunoblots for all patient samples were quantified and the fold change in protein is shown (right; normalized to GAPDH then displayed as fold change relative to the vehicle treatment). The red line indicated the average fold change for all 7 samples. The control lysate (Ctrl) is isolated from Mec1 B-cells. **d** XLA cell lines (BTK null) were transfected to express either wild type or C481S mutant BTK. Cell lines were then treated for 16 h with vehicle, 1uM ibrutinib (for 1 h followed by washout), or 100nM SNX-2112. Whole cell lysates were collected and immunoblot analysis performed for phospho-BTK and total BTK, as well as the loading control Actin. Immunoblots were quantified and the fold change in protein is shown below the graph (normalized to Actin then displayed as fold change relative to the vehicle). The control lysates are parental 293T cells (Ctrl -) and 293T cells over-expressing BTK protein (Ctrl +)

Next, we tested the efficacy of SNX-5422 in combination with ibrutinib using a Eμ-TCL1 CLL ibrutinib resistant model previously reported by our group [10, 11]. Mice dosed with vehicle or ibrutinib had similar overall survival, however the combination of SNX-5422 and ibrutinib provided remarkable survival benefit (Fig. 2a; 51 day median survival in the vehicle and ibrutinib groups versus 100 day median survival in the combination; *p* < 0.01). Mice treated with SNX-5422 had smaller spleens when compared to vehicle and ibrutinib treated mice (Fig. 2b, SNX-5422 + ibrutinib vs. vehicle or ibrutinib: *p* < 0.001), with a trend towards decreased peripheral blood tumor development (green line, Fig. 2c). Finally, histopathological analysis of leukemic infiltration into surrounding tissues revealed that mice treated with combination therapy had reduced severity of neoplastic infiltrates in liver, spleen, and lung (Fig. 2d). We performed an additional in vivo study using the Eμ-BRD2 model which develops an aggressive lymphoma with splenomegaly, abdominal lymphadenopathy and leukemic infiltrations of liver and lung [12]. While there was not a significant survival advantage (Additional file 1A) there was reduced splenic tumor burden in the SNX-5422 treated mice (Additional file 1B) and a complete absence of neoplasia in the SNX-5422 treated animals at time of death (Additional file 1C).

**Fig. 2:**
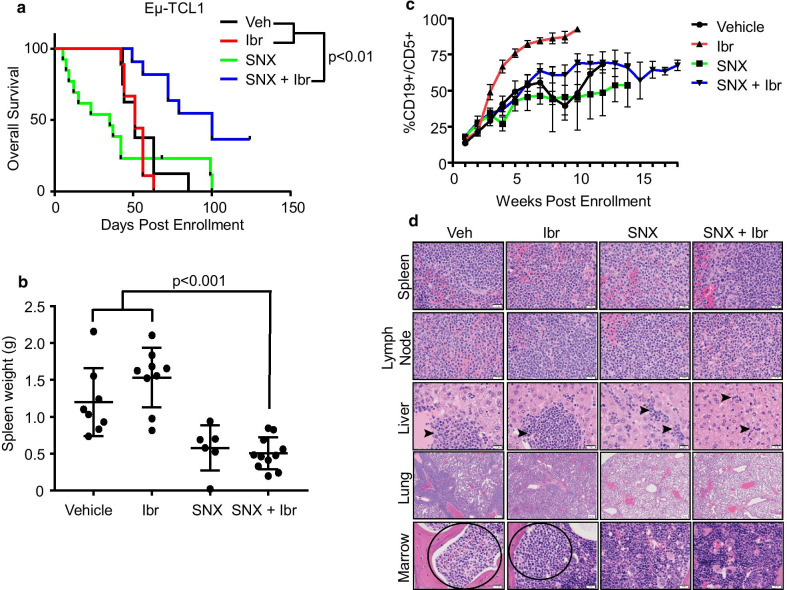
**a** Kaplan-Meier estimates of overall survival in mice engrafted with ibrutinib resistant TCL1 splenocytes and treated with vehicle, ibrutinib (30 mg/kg daily in drinking water), SNX-5422 (50 mg/kg 3 days/week) or the combination. The log-rank p-value between the combination SNX-5422 + ibrutinib vs either vehicle or ibrutinib was calculated (combo vs vehicle,* p* = 0.007; combo vs ibrutinib,* p* = 0.002). **b** Mice spleens were weighed at early removal criteria and compared between treatment groups (combo vs vehicle,* p* < 0.001; combo vs ibrutinib,* p* < 0.001; vehicle vs ibrutinib,* p* = 0.035). **c** Weekly peripheral blood disease burden evaluated by %CD19+CD5+ of CD45+ cells of mice described in Figure 3A and that are alive at each time point. **d** Histopathology performed on spleen, lymph node, liver, lung and bone marrow reveals reduced leukemic infiltration in the liver, lungs, and marrow of SNX-5422 and SNX-5422 + ibrutinib treated groups

We did note reduced survival in the single agent SNX-5422 treated mice despite the reduced tumor burden, therefore we performed detailed histopathology in both in vivo models. Surprisingly, there was marked ulceration of the non-glandular stomach (Additional file 2; indicated by black arrows) accompanied by immune cell infiltration (indicated by red arrows), and hyperplasia of the surrounding squamous gastric mucosa (indicated by green arrows) which was less severe in mice treated with the combination. As these ulcers only occurred in the non-glandular stomach (an anatomic counterpart lacking in humans), this toxicity is unlikely to occur in human patients receiving SNX-5422, which was safe and tolerable with a recommended dose of 100 mg/m^2^ QOD 3 weeks on and 1 week off in phase I solid tumor trials [5]. Interestingly, in both models these symptoms appear to be less severe in mice treated with the combination, suggesting that ibrutinib is ameliorating some of the toxicities related to SNX-5422.

Altogether, our data suggest that Hsp90 inhibition in combination with ibrutinib may be an option for initial treatment in CLL to prevent the outgrowth of a resistant clone in patients who display high risk features that are less likely to have a prolonged response to ibrutinib. The safety and pharmacology of SNX-5422 has been explored in clinical trials in solid tumors (NCT01611623) and hematological malignancies (NCT01635712). However, studies to determine the efficacy in CLL in patients receiving ibrutinib therapy (NCT02973399, NCT02914327) have not been completed due to low enrollment and have not been published. This is likely attributed to the overall success of ibrutinib and other BTK inhibitors, as well as newer generation BTK inhibitors that do not rely on the C481S binding site currently in development. Nevertheless our work shows that alternative strategies that target BTK for degradation are a promising option in BTK inhibitor resistant CLL.

## Supplementary Information

**Additional file 1.** Eμ-BRD2 in vivo model. **A** Kaplan–Meier estimates of overall survival in mice engrafted with ibrutinib resistant BRD2 splenocytes and treated with vehicle, ibrutinib (30 mg/kg daily in drinking water), SNX-5422 (50 mg/kg 3 days/week) or the combination. **B** Mice spleens were weighed at early removal criteria and compared between treatment groups. **C** Histopathology performed on spleen, lymph node, liver, lung and bone marrow reveals reduced leukemic infiltration in the liver, lungs, and marrow of SNX-5422 and SNX-5422 + ibrutinib treated groups. (C, lymph node cortex; M, lymph node medulla; W, spleen white pulp; R, spleen red pulp).

**Additional file 2.** SNX-5422 related toxicity. Histopathology of the murine non-glandular stomach reveals gastric ulcers in SNX-5422 treated groups in both the Eμ-TCL1 and the Eμ-BRD2 mouse models. The black arrows indicate the damage to the gastric mucosal layer in the SNX-5422 and combo treated mice, red arrows indicate immune cell infiltration, and green arrows indicate mucosal hyperplasia.

## Data Availability

All data collected during this study are included in this published article or the supplementary information.

## Abbreviations

CLL: Chronic lymphocytic leukemia
BCR: B-cell receptor
BTK: Bruton’s tyrosine kinase
XLA: X-linked agammaglobulinemia
