# Supplementary material for: Preclinical evaluation of the Hsp90 inhibitor SNX-5422 in ibrutinib resistant CLL
Source: J Hematol Oncol. 2021 Feb 24;14:36. doi: 10.1186/s13045-021-01039-9 (PMC7905592; doi:10.1186/s13045-021-01039-9)
Supplement: Supplementary file 1 — Additional file 1. Eμ-BRD2 in vivo model. A Kaplan–Meier estimates of overall survival in mice engrafted with ibrutinib resistant BRD2 splenocytes and treated with vehicle, ibrutinib (30 mg/kg daily in drinking water), SNX-5422 (50 mg/kg 3 days/week) or the combination. B Mice spleens were weighed at early removal criteria and compared between treatment groups. C Histopathology performed on spleen, lymph node, liver, lung and bone marrow reveals reduced leukemic infiltration in the liver, lungs, and marrow of SNX-5422 and SNX-5422 + ibrutinib treated groups. (C, lymph node cortex; M, lymph node medulla; W, spleen white pulp; R, spleen red pulp). [file 13045_2021_1039_MOESM1_ESM.pdf]

Supplemental Figure 1

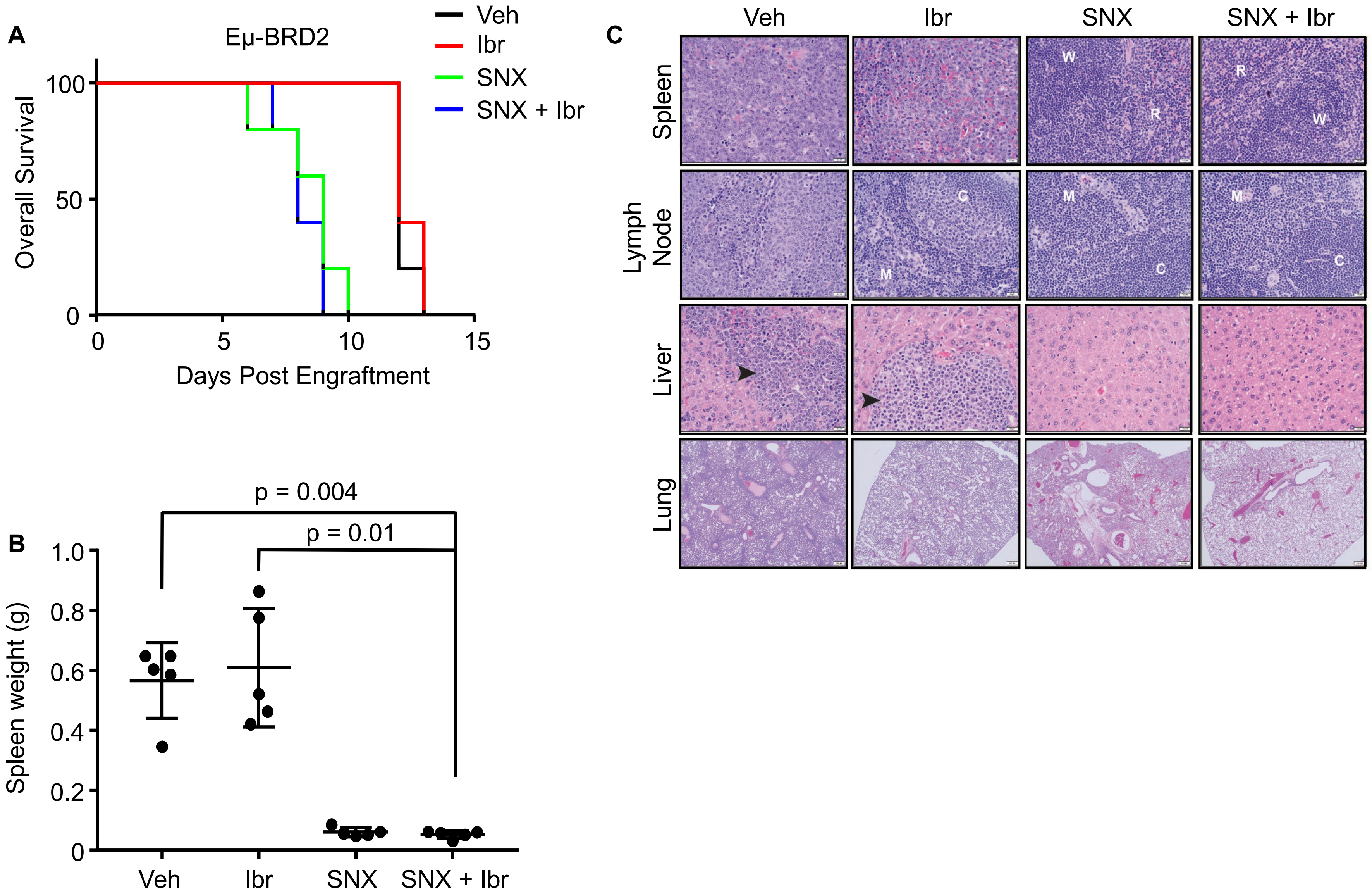

Supplemental Figure 1: A. Kaplan-Meier estimates of overall survival in mice engrafted with ibrutinib resistant BRD2 splenocytes and treated with vehicle, ibrutinib (30 mg/kg daily in drinking water), SNX-5422 (50 mg/kg 3 days/week) or the combination. **B.** Mice spleens were weighed at early removal criteria and compared between treatment groups. **C.** Histopathology performed on spleen, lymph node, liver, lung and bone marrow reveals reduced leukemic infiltration in the liver, lungs, and marrow of SNX-5422 and SNX-5422 + ibrutinib treated groups. (C, lymph node cortex; M, lymph node medulla; W, spleen white pulp; R, spleen red pulp).
