# Supplementary material for: Preclinical evaluation of the Hsp90 inhibitor SNX-5422 in ibrutinib resistant CLL
Source: J Hematol Oncol. 2021 Feb 24;14:36. doi: 10.1186/s13045-021-01039-9 (PMC7905592; doi:10.1186/s13045-021-01039-9)
Supplement: Supplementary file 2 — Additional file 2. SNX-5422 related toxicity. Histopathology of the murine non-glandular stomach reveals gastric ulcers in SNX-5422 treated groups in both the Eμ-TCL1 and the Eμ-BRD2 mouse models. The black arrows indicate the damage to the gastric mucosal layer in the SNX-5422 and combo treated mice, red arrows indicate immune cell infiltration, and green arrows indicate mucosal hyperplasia. [file 13045_2021_1039_MOESM2_ESM.pdf]

Supplemental Figure 2

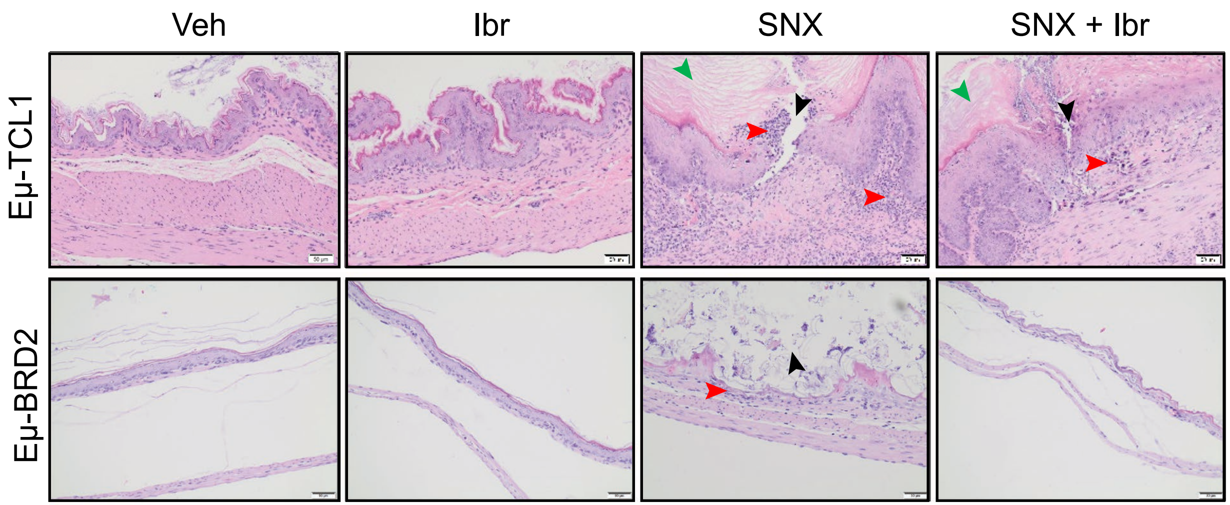

Supplemental Figure 2: Histopathology of the murine non-glandular stomach reveals gastric ulcers in SNX-5422 treated groups in both the Eμ-TCL1 and the Eμ-BRD2 mouse models. The black arrows indicate the damage to the gastric mucosal layer in the SNX-5422 and combo treated mice, red arrows indicate immune cell infiltration, and green arrows indicate mucosal hyperplasia.
